# Supplementary material for: Streptococcus pyogenes M1T1 Variants Induce an Inflammatory Neutrophil Phenotype Including Activation of Inflammatory Caspases
Source: Front Cell Infect Microbiol. 2021 Jan 28;10:596023. doi: 10.3389/fcimb.2020.596023 (PMC7876443; doi:10.3389/fcimb.2020.596023)
Supplement: Supplementary file 1 [file DataSheet_1.pdf]

Supplementary Material

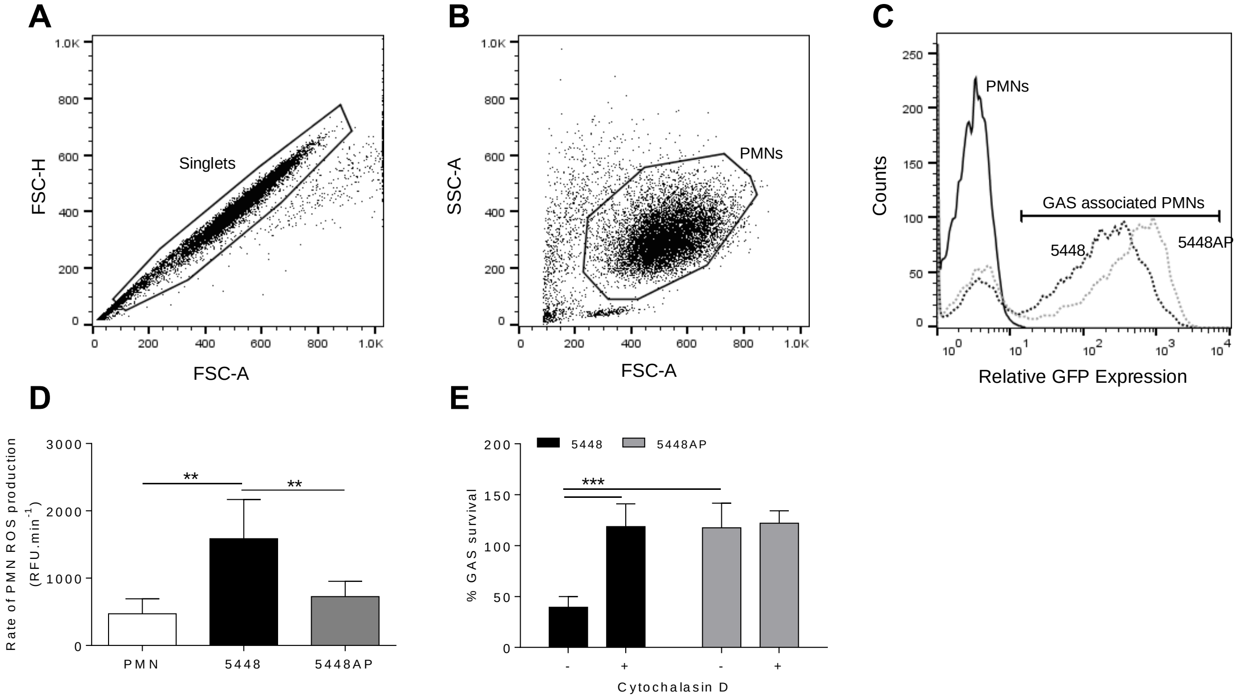

Supplementary figure 1. The association of fluorescent GAS (GFP) to human neutrophils determined via flow cytometry. (A) Primary neutrophil (PMN) 'Singlets' cell selection (FSC-A vs. FSC-H) and (B) viable 'PMNs' discrimination (FSC-A vs. SSC-A). (C) Representative histogram showing relative fluorescence (GFP) of GAS infected neutrophils and gating for the selection of GAS-neutrophil association. (D) Rate of ROS production by neutrophils between 30-60 min (n=6 donors). (E) GAS killing by human neutrophils is inhibited at 30 min following pre-incubation with actin polymerising molecule cytochalasin D (n=4 donors, Sidak's multiple comparison). Results are the pooled means±SD (of triplicate measurements for panels D and E). **p<0.01, ***p<0.001.

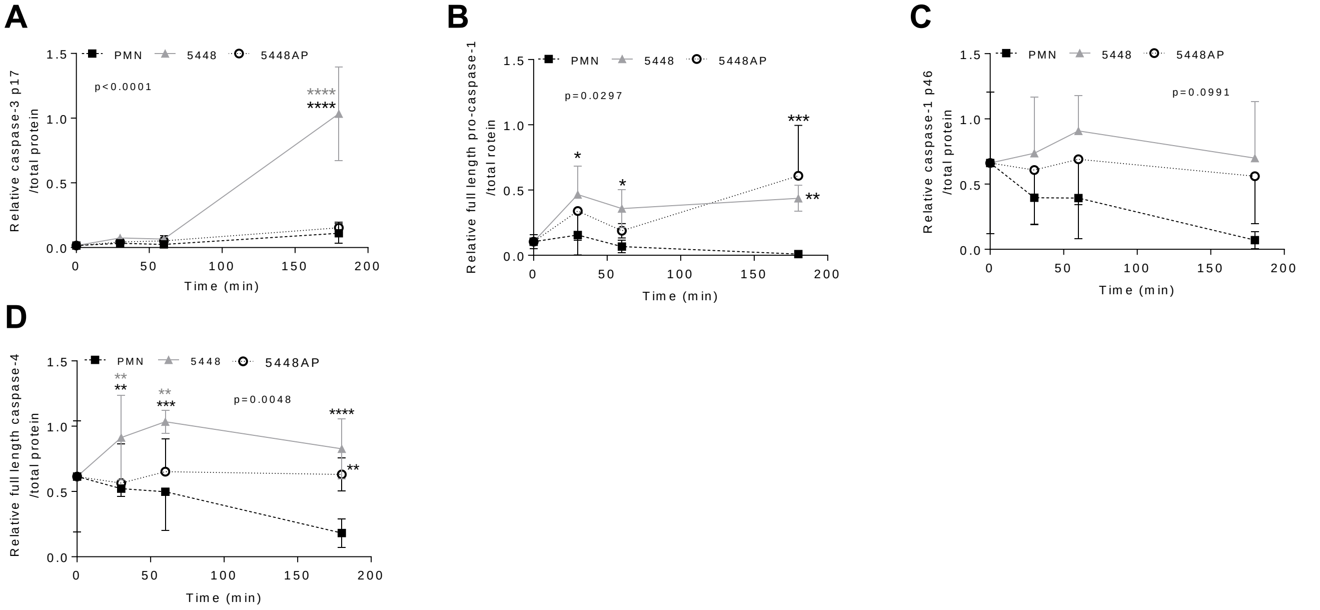

Supplementary figure 2. Quantification of protein bands in human neutrophil lysates identified through immunoblotting. (A) Caspase-3 p17, (B) caspase-1 full-length, (D) caspase-1 p46 and (D) caspase-4 immunoblots were imaged then analysed using ImageJ where area under the curve was normalised over total protein. Results are the means±SD where 3 donors were used. *p<0.05, **p<0.01, ***p<0.001 and ****p<0.0001, with black asterisks denoting significance to control and grey asterisks between 5448 and 5448AP.

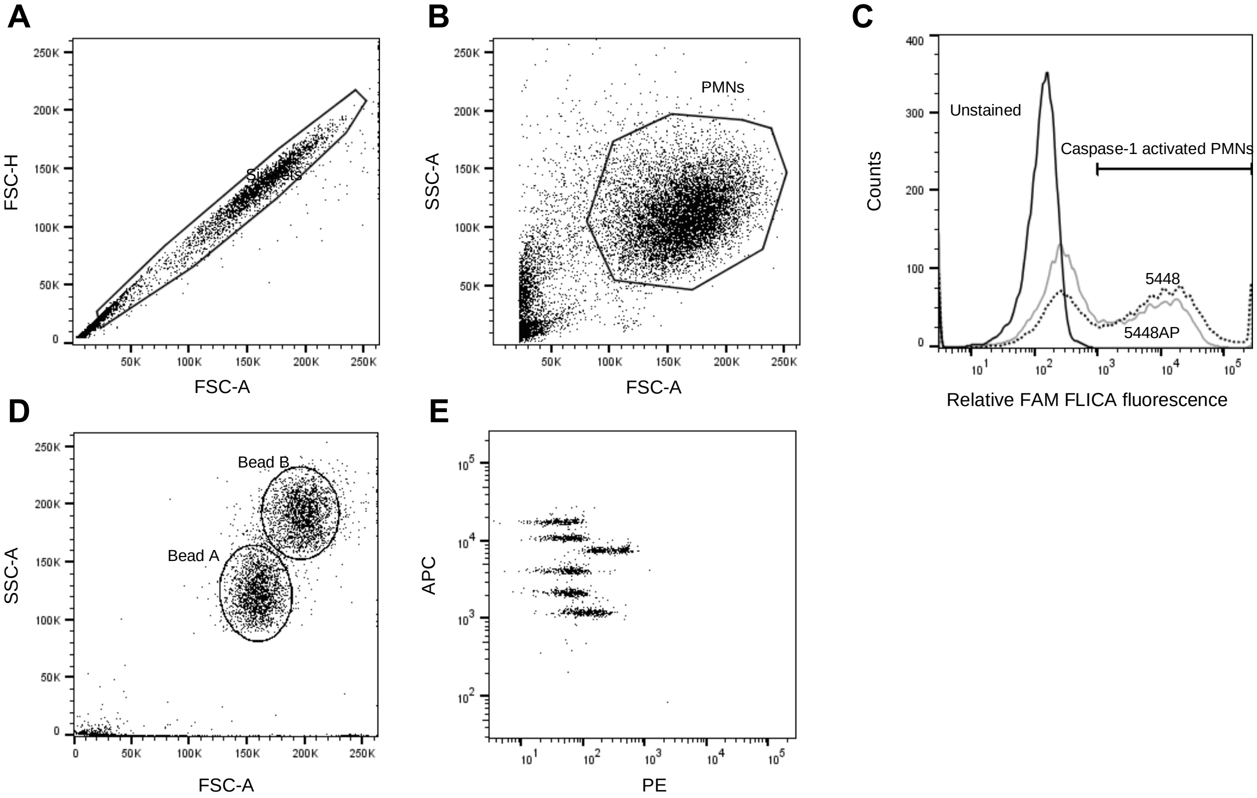

Supplementary figure 3. Human neutrophils were assessed for the activation of caspase-1 and release of cytokines via and flow cytometry. (A) Primary neutrophil (PMN) 'Singlet' cell selection (FSC-A vs. FSC-H) and (B) viable 'PMNs' discrimination (FSC-A vs. SSC-A). (C) Representative plot showing unstained and GAS infected neutrophils (Singlets/PMNs) with FLICA (FAM-YVAD-FMK) fluorescence. Gating strategy for LEGENDplex cytometric bead assay where (D) beads A and B were gated (FSC-A vs. SSC-A). Gates for beads A (shown) and B were assessed for (E) PE vs. APC fluorescence where cytokines had unique APC fluorescence and expression was measured via PE fluorescence when compared to a standard curve.

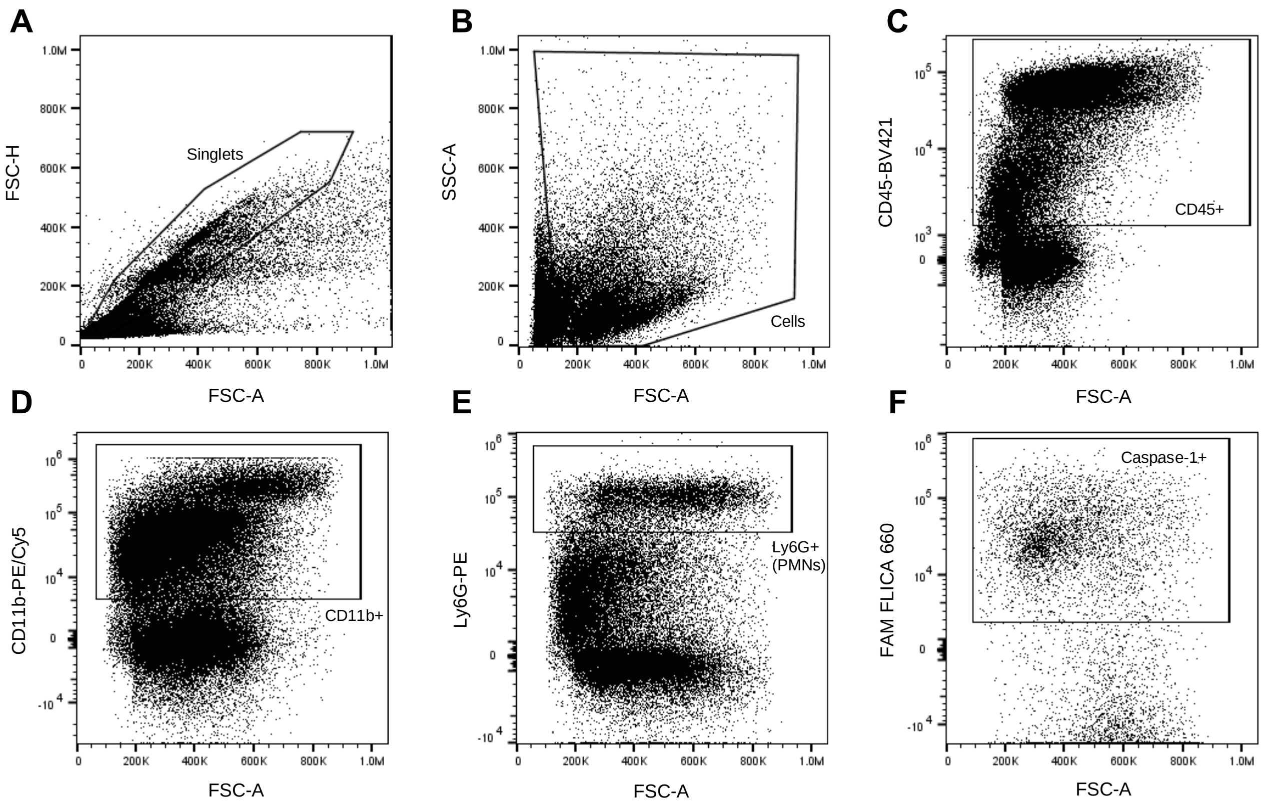

Supplementary figure 4. Flow cytometric sequential gating strategy for identification of murine neutrophils determining caspase-1 activation. (A) Primary 'Singlets' cell selection (FSC-A vs. FSC-H) and (B) 'Cells' discrimination (FSC-A vs. SSC-A) of murine blood and lavage fluid. 'Cells' were further gated upon (C) CD45-BV421 expression and (D) CD11b-PE/Cy5 expression. Neutrophils (PMNS) were defined as (E) Ly-6G-PE+ cells from the CD45+/CD11b+ population. Neutrophils were assessed for (F) caspase-1 activation using FLICA 660 (660-YVAD-FMK).
